# Impact of Clostridioides difficile Reporting on Antimicrobial Therapy Days Directed at Treatment of C. difficile Infections

**DOI:** 10.1017/ash.2024.184

**Published:** 2024-09-16

**Authors:** Ardath Plauche, Edward Septimus, Jamie Thomas

**Affiliations:** Memorial Hermann Health System; Harvard Medical School and Harvard Pilgrim Health Care Institute; Memorial Hermann Southwest

## Abstract

**Background:** Previous studies have found that solely relying on molecular testing is likely to result in the overdiagnosis and overtreatment of C. difficile infections (CDI). Comparable outcomes have been demonstrated in patients with a positive molecular test (C. difficile PCR) result and a negative toxin immunoassay (C. difficile toxin) compared to patients without CDI by either testing **Method:** In 2021 Memorial Hermann Healthcare System converted from C. difficile PCR testing only to C. difficile PCR testing with reflex to C. difficile toxin if positive. A previous internal audit revealed that despite this change in testing, patients who were C. difficile PCR positive and C. difficile toxin negative were still receiving treatment. This study aimed to evaluate the impact of C. difficile reporting on the total days of therapy directed at the treatment of CDI of an 11-hospital health care system in patients who testing C. difficile PCR positive/C. difficile toxin negative. **Methods:** Pre-post, multicenter, retrospective, observational study conducted from January 1, 2023 through March 31, 2023 (pre-intervention) and July 1, 2023 through September 31, 2023 (post-intervention) which included hospitalized adult patients with a C. difficile test ordered within the study period. Intervention included a change in reporting of C. difficile PCR positive/C. difficile toxin negative results to display a laboratory comment. The comment notifies providers of the positive C. difficile PCR result while highlighting this probably reflects colonization with C. difficile as the C. difficile toxin is negative and treatment is rarely indicated. **Results:** In total, 989 C. difficile PCR were order in the pre-intervention cohort compared to 1009 in post-intervention. The overall rate of patients that received therapy directed at CDI decreased from 14% to 10% after the implementation of reporting change. Total days of therapy (DOT) also decreased by 29% from 482 to 342. Days of therapy that were administered to patients with C. difficile PCR positive/negative C. difficile toxin test decreased from 183 to 91. **Conclusions:** Adjusting the reporting of C. difficile results led to an overall numerical decrease of antimicrobial DOT directed at CDI treatment. In particular, among patients with a positive C. difficile PCR/C. difficile toxin negative test a 50% reduction in DOT was observed. Further data are required to assess the overall clinical impact of adjusting CDI reporting methods.

